# Is Peri-Operative Steroid Replacement Therapy Necessary for the Pituitary Adenomas Treated with Surgery? A Systematic Review and Meta Analysis

**DOI:** 10.1371/journal.pone.0119621

**Published:** 2015-03-16

**Authors:** Mamatemin Tohti, Junyang Li, Yuan Zhou, Yuebing Hu, Zhuang Yu, Chiyuan Ma

**Affiliations:** Department of Neurosurgery, Jinling Hospital, School of Medicine, Nanjing University, Nanjing, China; Weill Cornell Medical College Qatar, QATAR

## Abstract

**Background:**

Patients with pituitary adenomas usually receive “stress dose” steroids in the peri-operative peroids. Though randomized controlled trials(RCT) have not been performed to assess the necessity of steroid coverage, there are several studies that explained the changes of adrenal function during peri-operative peroids. The aim of the present study is to investigate whether it is necessary to employ conventional peri-operative glucocorticoid replacement therapy to all the patients undergoing surgery.

**Methods:**

We searched studies addressing peri-operative steroids coverage for pituitary adenomas in the Web of Science, Medline and the Cochrane Library. Then we extracted studies about peri-operative morning serum cortisol(MSC) levels, morbidity of early postoperative adrenal insufficiency, postoperative diabetes insipidus, relationships between MSC levels and adrenal integrity. We used RevMan Software to combine the results for meta-analysis. We used fixed-effects models for there was no significant heterogeneity existed.

**Findings:**

There are 18 studies from 11 countries published between 1987 and 2013 including 1224 patients. The postoperative serum cortisol levels were significantly increased compared with the preoperative one in hypothalamic-pituitary-adrenal axis(HPAA) functions preserved patients(P<0.00001). The morbidity of early postoperative adrenal insufficiency ranged from 0.96% to 12.90%, with the overall morbidity of 5.55%(41/739). There was no significant differences of early postoperative diabetes insipidus between no supplementation patients and in supplementation patients(P=0.82). Conversely, there may be some disadvantages of high levels of cortisols such as high incidence of osteopenia and bone derangement and even the increased mortality rate. The patients with MSC levels of less than 60 nmol/l at 3 days after operation is considered as adrenal insufficient and more than 270 nmol/l as adrenal sufficient. To patients with MSC levels of 60–270 nmol/l, we need more clinical data to establish further cortisol supplementation criteria.

## Introduction

Pituitary adenomas makes up 10%~15% of intracranial tumors. Over the last four decades, the preferred treatment to pituitary adenomas has been the transsphenoidal surgery(TSS) for its obvious advantages such as the quick relief of signs and symptoms, the arrest of permanent damage to organ systems caused by the hormonal excess[1].

Patients undergoing TSS usually receive “stress dose” steroids whether the hypothalamic-pituitary-adrenal axis(HPAA) is deficient or preserved during TSS[2]. Though randomized controlled trials(RCT) have not been performed to assess the necessity of steroid coverage[3], there are several studies on the changes of HPAA functions associated with pituitary surgery. Until now, some clinical data revealed that there was no necessity for steroid supplementation in HPAA preserved patients during TSS[4, 5].

The aim of present study is to investigate whether it is necessary to employ conventional peri-operative glucocorticoid replacement therapy to all patients by analyzing peri-operative cortisol changes in serum of patients undergoing TSS. We also discussed the criteria for postoperative HPAA integrity which was essential for appropriate endocrine management by measuring the morning serum cortisol(MSC) levels.

## Materials and Methods

### Search strategy and selection criteria

The following databases from which studies addressing the peri-operative steroid coverage for pituitary adenomas were searched: Web of Science(up to December 2013), Medline(up to December 2013), the Cochrane Library(up to December 2013). The key words were described in supporting material(S1 File). We searched the reference lists of all relevant publications for additional studies. In cases where multiple publications existed from the same study, we included the article with more information.

We did not exclude any article on the basis of language. However, we excluded studies about the Cushing disease for its specificity. We also excluded studies that were not original, studies that did not report the outcomes of our interests, studies whose full text could not be obtained, studies with insufficient data for meta-analysis and studies with less than 5 patients.

MT did the literature search, screening of the abstracts and full texts for eligible studies. To assess further eligibility of the studies, JYL and YBH independently assessed the full texts according to the predefined inclusion criteria. If a full text was judged by both authors to meet the requirements, the article was included. Any disagreement was resolved by a third reviewer (CYM). In all cases, this verification was consistent with the majority view of the reviewers. Then we selected 18 studies[4–21].

### Data extraction

After our initial assessment for eligibility, MT and JYL independently completed the data extraction, any disagreement about the data was resolved by CYM. The following data were extracted: 1) study subjects: first author, years, number of subjects, gender distributions, ages, type of the tumors, size of the tumors, observation and follow-up periods; 2) study design: open label, prospective, retrospective, randomized or blinded; 3) study data: peri-operative cortisol levels(if there were more than one postoperative morning cortisol level data, we selected the minimum one), steroid types, administeration periods(preoperatively, intra-operatively or postoperatively), dosage administered, routines of administeration, clinical features associated with HPAA insufficiency (symptoms of fatigue, loss of appetite, nausea, vomiting, arthralgias, and, in some instances, hypotension, electrolyte abnormalities[16]), morbidity of transient/permanent diabetes insipidus, and other clinical features mentioned in these studies; 4) HPAA function assessment data: tests to assess the HPAA functions, assessment peroids, dosage of drugs, administeration peroids, routines of administeration, cortisol detection peroids, HPAA assessment standards, number of HPAA sufficient and insufficient subjects.

On the basis of this study, we subdivided the studies according to our research objects. First of all, we selected the studies about peri-operative MSC levels. We selected studies that preoperative MSC levels were in normal range and did not use the peri-operative cortisols. Then the postoperative MSC levels were compared with the preoperative one. Secondly, we classified studies of patients who need steroid supplementation at the early postoperative peroids and studies about the morbidity of postoperative diabetes insipidus. In studies about adrenal insufficiency, we briefly calculated the morbidity of adrenal insufficiency in patients who had normal preoperative HPAA functions. In studies about diabetes insipidus, we selected the patients who had administered the peri-operative cortisol as control group. Finally, we briefly sorted out the relationship between the MSC levels and HPAA integrity using the diagnostic evaluation data in 9 studies to choose the most suitable criterion for adrenal insufficiency/sufficiency[6, 7, 10–13, 15–17].

### Statistical analysis

The results were expressed as the mean(SD), median and range, absolute value and percentage. The odd ratio(OR) was used as a measure of association, with its 95% confidence interval. The Revman Software5.2(The Cochrane Collaboration, 2008) was used for meta-analysis. In consistency of studies (study-to-study variation) was assessed using the x2-statistic (the hypothesis tested was that the studies are all drawn from the same population, i.e. from a population with the same effect size). Fixed effects models were used for there was no significant heterogeneity existed.

## Results

There were 18 studies from 11 countries that met our predefined inclusion criteria(S1 Fig.), 7 is retrospective and 11 is prospective(Table 1). All of them were published from 1987 to 2013 including 1224 patients. Most of these patients had been followed up for a period of time, from one week to 3.5 years. Cushing disease was excluded from these studies for its specificity, for the majority were GH secreting, PRL secreting and non-functioning pituitary adenomas.

**Table 1:** Overview of 18 included studies.

Author+years+reference	Study design	Country	Years of study	Patients included	Male(%)	Age distribution	Macroadenoma(%)	GH	PRL	NF	Others	Follow-up peroids
**Hagg 1987[10]**	Retrospective	Sweden	1977–1983	68	30(44.1%)	18–84(M:42)	*	*	*	*	*	1–4 weeks
**Hout 1988[4]**	Prospective	USA	-1988	88	12(13.6%)	16–72(M:32.5)	49(55.7%)	3	71	9	4	3 months
**Garcia-Luna 1990[5]**	Prospective	Spain	-1990	10	3(30%)	21–54(M:38)	8 (80%)	4	2	4	0	2 years
**Page 1994[18]**	Retrospective	UK	-1994	31	*	*	*	*	*	*	*	1 months
**auchus 1997[6]**	Prospective	USA	-1997	28	14 (50%)	25–71(M:43.8)	6 (21.4%)	10	6	4	8	2–20 months
**Courtney 2000[7]**	Prospective	UK	-2000	42	21 (50%)	23–73(M:49)	*	7	5	29	1	4–6 weeks
**Rajaratnam 2003[19]**	Prospective	India	-2003	130	*	16–66(M:40)	130(100%)	18	28	48	36	6 months
**Klose 2005[13]**	Retrospective	Denmark	1992–2001	110	66(58.2%)	50.9±15.7	98 (89.1%)	31	10	66	3	12 months
**Kristof 2008[14]**	Prospective	Germany	-2008	37	16 (43.2%)	50.6±20	37(100%)	*	*	*	*	12 weeks
**Wentworth 2008[21]**	Prospective	Australia	2004–2006	56	34(60.7%)	19–82(M:50)	52 (92.9%)	8	6	39	3	6–24 month
**Cozzi 2009[8]**	Prospective	Italy	-2009	72	37(51.4%)	20–87	72(100%)	0	0	72	0	12 months
**Jayasena 2009[11]**	Retrospective	UK	2001–2008	36	22(61.1%)	19–69(M:44)	23(63.9%)	13	0	13	10	4–6 weeks
**Marko 2009[15]**	Prospective	USA	2003–2005	83	46 (55.4%)	*	75(90.4%)	*	*	*	*	1–3 months
**Kraca 2010[12]**	Prospective	Turkey	-2010	64	32 (50%)	18–72(M:43)	*	28	10	25	1	1 months
**Marko 2010[16]**	Prospective	USA	2006–2009	100	56(56%)	53.6±15.1	*	12	9	81	2	7–42 month
**De Tommasi 2012[9]**	Retrospective	Canada	2000–2011	45	23 (51.1%)	18–84(M:51)	*	18	0	26	1	1–18 months
**McLaughlin 2013[17]**	Retrospective	USA	2007–2010	139	76(54.7%)	17–88(M:50)	119(85.6%)	21	35	80	3	3–41 months
**Regan 2013[20]**	Retrospective	USA	2007–2012	85	59(69.4%)	53.2±14.7	*	15	3	67	1	2 weeks

**Abbreviation**: M(Age distribution column): Mean age; GH: Growth Hormone secreting adenomas; PRL: Prolactin secreting adenomas; NF: Non functioning adenomas

There were 7 studies involving the peri-operative serum cortisol levels in pituitary adenomas patients who had normal serum cortisol levels and no clinical manifestations of adrenal insufficiency preoperatively[4, 5, 8, 9, 14, 19, 21]. They were published from 1988 to 2012 including 230 pituitary adenomas patients. There were 3 studies whose postoperative serum cortisol levels were lower than preoperative levels[4, 5, 19] and 4 were the opposite[8, 9, 14, 21]. From the forest plot(S2 Fig.), we concluded that the postoperative cortisol levels increased significantly compared with the preoperative one in preoperative HPAA functions preserved patients(P<0.00001). The Mean Difference(IV, Fixed, 95% CI) was 30.86 [21.68, 40.08].

For the early postoperative incidence of adrenal insufficiency, we briefly summarized the morbidity of adrenal insufficiency in the preoperative HPAA integrated patients(Table 2) for the absence of RCT. There were 12 studies meeting our inclusion criteria[4, 5, 8, 9, 13–18, 20, 21]. The morbidity of early postoperative adrenal insufficiency ranged from 0.96% to 12.90%, with the overall morbidity of 5.55%(41/739).

The early postoperative diabetes insipidus was mentioned in 3 studies with 235 patients[5, 9, 19, 20]. There was no significant difference of early postoperative diabetes insipidus in two groups, while the morbidity was 4 in no supplementation patients vs 12 in supplementation patients(P = 0.82). The Odds Ratio(M-H, Fixed, 95% CI) was 1.18[0.29, 4.80] (S3 Fig.).

**Table 2:** The morbidity of postoperative early adrenal insufficiency in patients with normal preoperative MSC levels.

Study+year+reference	Total patients	Events	Morbidity
**Hout 1988[4]**	83	2	2.41%
**Garcia-luna 1990[5]**	10	1	10.00%
**Page 1994[18]**	31	4	12.90%
**Klose 200513]**	71	7	9.86%
**Kristof 2008[14]**	22	2	9.09%
**Wentworth 2008[21]**	44	3	6.82%
**Marko 2009[15]**	64	1	1.56%
**Cozzi 2009[8]**	58	6	10.34%
**Marko 2010[16]**	104	1	0.96%
**De tommasi 2012[9]**	27	2	7.41%
**Regan 2013[20]**	86	3	3.49%
**McLaughlin 2013[17]**	139	9	6.47%
**Total**	739	41	5.55%

There were 9 studies which mentioned the relationships between the MSC levels and HPAA integrity[6, 7, 10–13, 15–17]. The insufficiency criteria ranged from 60 nmol/l to 220 nmol/l, which were measured between the day of surgery and 14 days after surgery. The sufficiency criteria also differed from 111 nmol/l to 496 nmol/l. The HPAA integrity tests employed in these studies were the insulin tolerance test(ITT) in 7 studies, short synacthen test(SST) in 3 studies, cortrosyn stimulation test(CST) in 2 studies, metyrapone test(MTT) in one study. The time of visit varied between 4 weeks and 41 months. Different criterion in different peroid matched different specificity and sensitivity(Table 3, S4 Fig.). We chosen the MSC level of 60 nmol/l at 3 days after surgery for adrenal insufficiency criterion and 270 nmol/l for adrenal sufficiency criterion for their 100% specificity and high sensitivity(100% and 94% respectively).

**Table 3:** The predictive value of early postoperative MSC levels for HPAA integrity assessment.

author+year+reference	HPAA integrity test	Time of visit	Insufficiency criteria	Time	Specificity	Sensitivity	Sufficiency criteria	Time	Specificity	Sensitivity
**Hagg 1987[10]**	ITT	*	*	*	*	*	200 nmol/l	random	94%	86%
**Hagg 1987[10]**	ITT	*	*	*	*	*	300 nmol/l	random	94%	67%
**Auchus 1997[6]**	ITT	6–30 months	60 nmol/l	day3	100%	100%	270 nmol/l	day3	100%	94%
**Courtney 2000[7]**	ITT	4–6 weeks	100 nmol/l	day6–7	73%	100%	450 nmol/l	day6–7	100%	47%
**Courtney 2000[7]**	ITT	4–6 weeks	*	*	*	*	250 nmol/l	day6–7	100%	73%
**Klose 2005[13]**	SST/ITT	6–60 weeks	100 nmol/l	day4–14	100%	14%	450 nmol/l	day4–14	9%	93%
**Jayasena 2009[11]**	ITT	6 weeks	100 nmol/l	day5	100%	44%	450 nmol/l	day5	100%	45%
**Marko 2009[15]**	SST/ITT	1–3 months	*	*	*	*	415 nmol/l	day1–2	66.7%	80.5%
**Marko 2009[15]**	SST/ITT	1–3 months	*	*	*	*	332 nmol/l	day1–2	50%	85.7%
**Kraca 2010[12]**	ITT	1 month	193 nmol/l	day2	100%	27.3%	*	*	*	*
**Kraca 2010[12]**	ITT	1 month	220 nmol/l	day3	100%	21%	*	*	*	*
**Kraca 2010[12]**	ITT	1 month	193 nmol/l	day4	100%	27%	*	*	*	*
**Kraca 2010[12]**	ITT	1 month	165 nmol/l	day5	100%	26.9%	*	*	*	*
**Kraca 2010[12]**	ITT	1 month	83 nmol/l	day6	100%	17%	*	*	*	*
**Marko 2010[12]**	CST	4–6 weeks	*	*	*	*	415 nmol/l	day1	*	98%
**McLaughlin 2013[17]**	CST/MTT	3–41 months	*	*	*	*	111 nmol/l	day1–2	57%	96%

Abbreviation: ITT: Insulin tolerance test; SST: Short synacthen test; CST: Cortrosyn stimulation test; Time:Time when the serum cortisol levels measured.

## Discussion

In order to assess the HPAA integrity in peri-operative peroids for the need of glucocorticoid replacement therapy, we desire a simple, reliable evaluation method. MSC is a mostly used, simplest and the first line test to evaluate it[3, 8, 11, 12, 15–17]. However, there are some disagreements about the consideration of normal values. Hout et al. set the MSC level in normal subjects during the preoperative peroid as 138–552 nmol/l and Garcia-Luna established it as 165–635 nmol/l[4, 5]. Rajaratnam et al. concluded it as 193–690nmol/l and Kristof et al. as 220–690 nmol/l[14, 19]. Nonetheless, all of these authors reached a consensus that there was no any symptom of adrenal insufficiency such as fatigue, anorexia, nausea, vomiting, fever, hypotension, electrolyte and metabolic derangements during this peroid.

In present study, we found that there was significant increase of MSC levels during the postoperative peroids in 230 consecutive patients who had intact HPAA preoperatively(P<0.00001). This was consistent with another study by Regan et al. that had not been selected for its high heterogeneity[20]. In their study, the postoperative MSC levels increased significantly in most of patients with the mean levels from 276.8±155.1 nmol/l to 637.6±408.5 nmol/l. So we concluded that, most of the patients who had intact HPAA preoperatively didn’t necessarily need peri-operative cortisol replacement therapy.

For the morbidity of adrenal insufficiency, there was no any RCT comparing the morbidity of postoperative adrenal insufficiency in the cortisol supplementation groups with non-supplementation groups. Here we briefly summarized the early postoperative adrenal insufficiency morbidity of patients with normal preoperative cortisol levels. The results suggested that there was a low incidence of adrenal insufficiency with average morbidity of 5.55%(0.96%~12.90%). This may suggests the unnecessity of routine cortisol supplementation therapy.

Diabetes insipidus is a common problem encountered following transsphenoidal pituitary surgery[19]. For the morbidity of postoperative diabetes insipidus, there also existed some disagreements. Aubrey et al. found that the exogenous steroid supplementation might inhibit antidiuretic hormone (ADH) release and precipitated diabetes insipidus[22]. Udelsman et al. concluded that there was no apparent advantage in supraphysiological glucocorticoid prophylaxis during surgical stress in primates[23]. Our study demonstrated that there were no significant difference in the morbidity of diabetes insipidus between patients with cortisol replacement and no replacement therapy.

Some publications also reported the complications associated with exogenous steroid usage in the postoperative peroids. Agha et al. illustrated that glucocorticoid usage of inpatient made postoperative assessment of the HPAA function difficult. Okinaga et al. and Peacy et al. reported that high dose of cortisols were in direct proportion to high incidence of osteopenia and bone derangement[24, 25]. Zueger et al. revealed that there was a significant increase of mortality in three different cortisol dose groups from low to high(9.4%, 16.7% and 57.9% respectively)[26]. Other studies reported that high dose of cortisol replacement therapy was associated with unfavorable metabolic profile, higher intraocular pressure levels[27, 28]. These findings were in some respect consistent with the finding of Aubrey et al. that high doses of cortisols may increase the morbidity of diabetes insipidus[22]. So to the patients with sufficient cortisol levels, there indeed are no need to receive exogenous cortisols.

Accurate prediction of HPAA function after TSS is also essential for proper postoperative management in patients with pituitary adenomas. Studies of cortisol levels during the week following surgery suggest that MSC levels > 250–635 nmol/l at 3–7 days after operation may be predictive of normal long-term HPAA functions[6, 7, 29, 30]. For there was no consistency about the evaluation of adrenal functions, we chose the MSC level of 60 nmol/l at 3 days after operation as adrenal insufficiency criterion and 270 nmol/l as adrenal sufficiency criterion due to their 100% specificity and highest sensitivity(100% and 94% respectively). To patients with MSC levels of 60–270 nmol/l, we may need more clinical studies to establish further cortisol supplementation criteria. However, there are many factors influencing the results such as racial diversity, age differences and gender differences. Thus it can’t be the unique cortisol supplementation criterion for everyone.

As for the supplementation dosage of steroids to adrenal insufficient patients, there also existed many unconformities. Page et al. referred their protocol as hydrocortisone(HC) 30 mg/day and doubled on the day of operation[18]. Kristof et al. recommended the supplementation protocol at their institution for HPAA function impaired patients as 100 mg HC on the day of surgery, 80 mg HC on day1, 60 mg HC on day2, 50 mg HC on day 3, 35 mg HC on day 4, 25 mg HC on day 5, and, depending on clinical assessment, 15–25 mg HC on day 6–10[14]. However, many institutions implemented the protocol introduced by Inder and Hunt in 2002[11, 20, 21]. According to that protocol, the adrenal functions of patients were evaluated using the ACTH 1–24 (Synacthen) test(SST) together with MSC levels based on the results of some previous studies[4, 6, 7]. The whole protocol was introduced in detail at S5 Fig. So far, this may be one of the most detailed and widely implemented protocol for TSS patients.

## Limitation

For there is no RCT on the necessity of peri-operative cortisol replacement for pituitary surgery, we don’t have enough data to proof whether they have to implement the supplementation therapy[4]. There is no sufficient studies about comparing the complications between low dose group and no replacement group. We also don’t have enough population to enhance the precision of our conclusion. There are many inconsistencies about the normal MSC level for HPAA sufficient patients. The follow up periods are also different, varies from 1 week to 42 weeks.

## Conclusion

In present systematic review and meta analysis, we found that the serum cortisol levels were significantly increased in patients after TSS surgery. There was also no significantly increased postoperative adrenal insufficiency and diabetes insipidus in no supplementation group than in supplementation group. Conversely, there may be some disadvantages of high levels of cortisols such as high incidence of osteopenia and bone derangement and even the increased mortality rate. The patients with MSC levels of less than 60 nmol/l at 3 days after operation are considered as adrenal insufficient and more than 270 nmol/l as adrenal sufficient. To patients with MSC levels of 60–270 nmol/l, more clinical studies should be implemented to establish further cortisol supplementation criteria. In a word, there is indeed no necessity to receive routine cortisol replacement for patients with normal MSC levels and it may be used when the symptoms occur.

## Supporting Information

S1 Checklist PRISMA Checklist. (DOC)

S1 File The key words for searching from databases. (DOC)

S1 Fig Selection of included studies. (TIF)

S2 Fig Peri-operative cortisol levels in preoperative HPAA integrated patients. (TIF)

S3 Fig The early postoperative diabetes insipidus morbidity in patients with peri-operative cortisol supplementation vs no cortisol supplementation. (TIF)

S4 Fig The predictive value of early postoperative MSC levels for HPAA integrity assessment. (TIF)

S5 Fig Glucocorticoid replacement protocol for transsphenoidal pituitary adenomectomy in non-ACTH-secreting pituitary adenomas by Inder and Hunt. (TIF)
