# Supplementary figures and images for: Is Peri-Operative Steroid Replacement Therapy Necessary for the Pituitary Adenomas Treated with Surgery? A Systematic Review and Meta Analysis
Source: PLoS One. 2015 Mar 16;10(3):e0119621. doi: 10.1371/journal.pone.0119621 (PMC4361329; doi:10.1371/journal.pone.0119621)

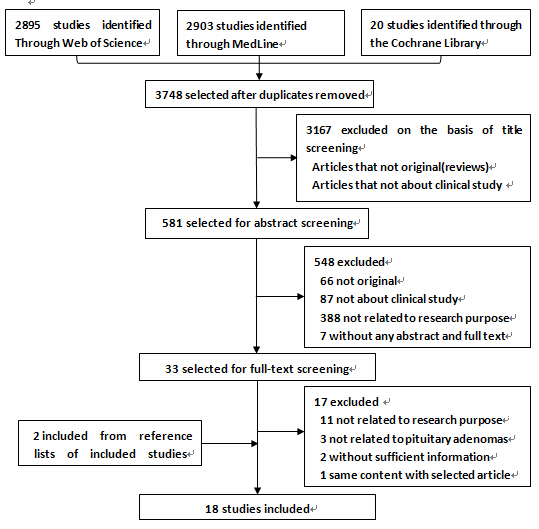

Supplement: S1 Fig — (TIF) [file pone.0119621.s003.tif]

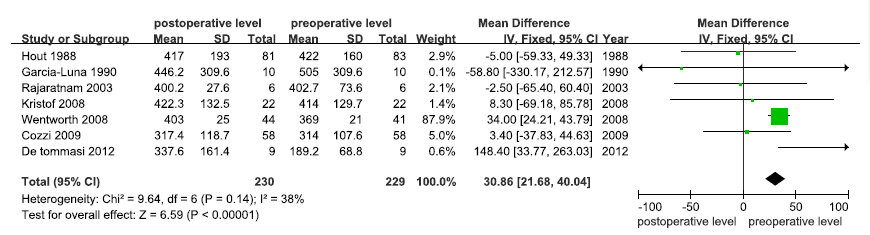

Supplement: S2 Fig — (TIF) [file pone.0119621.s004.tif]

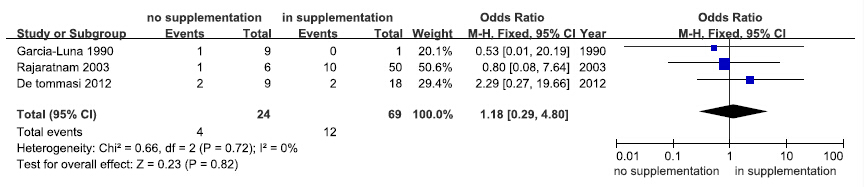

Supplement: S3 Fig — (TIF) [file pone.0119621.s005.tif]

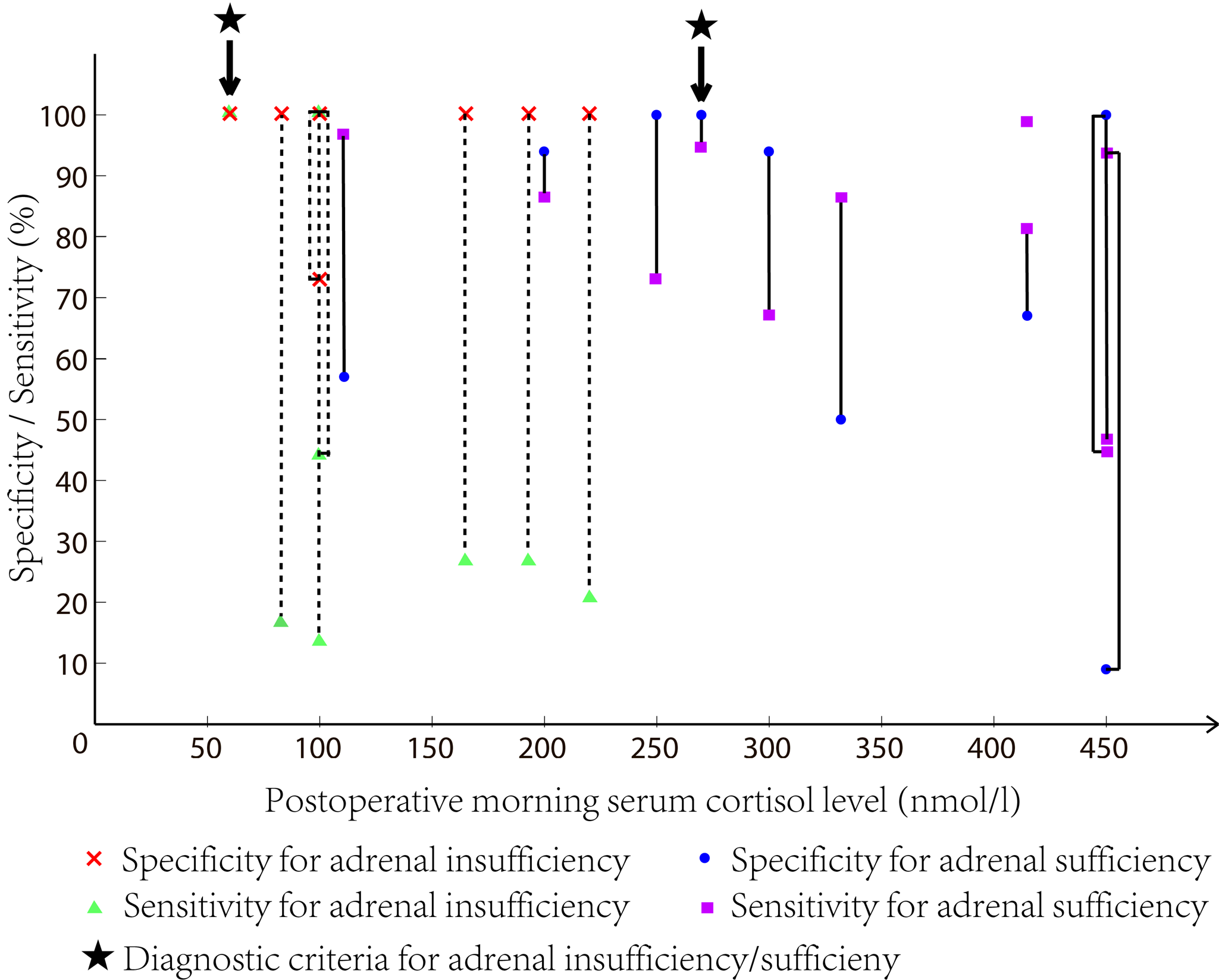

Supplement: S4 Fig — (TIF) [file pone.0119621.s006.tif]

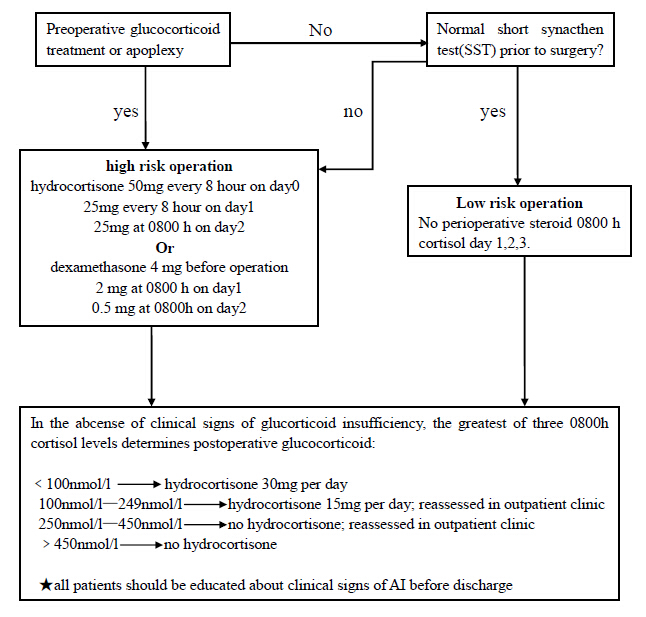

Supplement: S5 Fig — (TIF) [file pone.0119621.s007.tif]
